# Assessment of reliability and information quality of YouTube videos about root canal treatment after 2016

**DOI:** 10.1186/s12903-022-02540-4

**Published:** 2022-11-16

**Authors:** Myoung-jun Jung, Min-Seock Seo

**Affiliations:** Department of Conservative Dentistry, Wonkwang University Daejeon Dental Hospital, 77 Dunsan-ro, Seo-gu, Daejeon, 35233 South Korea

**Keywords:** Root canal treatment, Information quality, Reliability, Root canal treatment, YouTube

## Abstract

**Background:**

This study aimed to assess and update the content, reliability, and information quality of content related to root canal treatment (RCTx) on YouTube and evaluate the correlation between each evaluation index.

**Methods:**

YouTube was searched using two terms related to RCTx (“root canal and endodontic treatment”). A total of 240 videos (120 for each search term) were screened. Exclusion criteria were as follows: no sound or visuals, non-English, irrelevant to the search term, longer than 15 min, duplicate, or old (uploaded before 2016). After exclusion, 50 videos of “root canal treatment” and 45 videos of “endodontic treatment” were analyzed. Video length, total number of views, likes, dislikes, comments, and days since upload were recorded using descriptive video data. Viewers’ interaction, reliability and information quality of the video, and quality of video content were measured using nondescriptive video data. The interaction index and video power index were used for viewer interactions, and the modified DISCERN index, JAMA criteria, and Global Quality Score were used to assess the reliability and information quality of the video. The quality of the video content was measured using the completeness score.

**Results:**

The videos of the “root canal treatment” group had a significantly higher completeness score for the etiology and symptoms (*p* < 0.05), and videos of the “endodontic treatment” group showed a higher interaction index, completeness score for the procedure (*p* < 0.05). Videos for dentists had significantly higher completeness scores for the procedure, while videos for laypersons had higher completeness scores for etiology, anatomy, symptoms, and prognosis (*p* < 0.05). Furthermore, the total completeness score and the interaction index of the videos for laypersons were significantly higher (*p* < 0.05). The videos uploaded by the university had a significantly higher modified DISCERN index (*p* = 0.044), and the JAMA score was significantly higher in the commercial group (*p* = 0.001).

**Conclusions:**

Although the accuracy of videos related to RCTx was higher in videos by universities and professionals, the total completeness of YouTube videos was low regardless of the video source. Therefore, professionals should be responsible for providing more accurate and reliable videos.

## Background

Conventionally, health-related information is provided through direct communication with health care providers. However, with the growth of information technology, access to the Internet has become easier than in the past, and as a result, 80% of Internet users obtain medical or dental information through online searches [1].

YouTube is the most widely used content hosting site where users can freely upload videos, and is the second most visited website after Google [2]. YouTube videos are played more than 5 billion times daily, with an average viewing time of at least 15 minutes a day. Every minute, more than 500 h of new content are uploaded to YouTube [3]. Importantly, YouTube videos can be uploaded without exact verification and can be accessed by anyone with an account. Therefore, videos must be evaluated because inaccurate or misleading information can be provided. There are several video evaluation tools to assess the reliability and educational quality, such as the modified DISCERN, Journal of American Medical Association (JAMA) score, and Global Quality Score (GQS).

Root canal treatment (RCTx), a very common dental procedure, preserves natural teeth by removing bacteria and cleaning the infected root canal to prevent reinfection. It is difficult to know how many root canal treatments are performed in actual dental procedures, but data from the National Health Insurance Company indicate that RCTx is performed on hundreds of thousands of teeth every year in the USA [4]. As the procedure is practiced considerably, there is no doubt that many people will search for this purpose on YouTube. Several studies have evaluated YouTube videos in the field of endodontics, such as instrument separation, pulpotomy, and pulp capping [5, 6]. One study evaluated the completeness of RCTx videos uploaded to YouTube for patients [7], but no studies have assessed the popularity, reliability, and quality of videos regarding RCTx for dentists and laypersons. Additionally, ongoing video evaluation is essential, as many YouTube videos are uploaded daily.

Therefore, the purpose of this study was to assess and update the reliability and information quality of content related to the RCTx on YouTube and to evaluate the correlation between each scoring tool.

## Methods

### Video selection

On November 13, 2021, we searched YouTube for relevant videos using two related search terms: root canal treatment and endodontic treatment. The search was carried out by deleting cookies and caching with Google Chrome, using the default settings without filters. Previous studies have shown that 90% of people watch the top 60 videos several times a day [2, 8]; the top 120 videos were selected and screened according to each search term (a total of 240 videos).

Since the number of YouTube users has increased significantly compared to 2016 when a previous study assessing YouTube content on RCTx was reported [7], videos uploaded after 2016 were chosen. The exclusion criteria were as follows: no sound or visuals, non-English, irrelevant to the search term, longer than 15 min, duplicate, or old (uploaded before 2016). Previous studies have shown that videos longer than 15 min are less likely to attract YouTube users [5, 6, 9], and videos shorter than 15 min were selected. Ethical committee approval was waived, as this study was conducted using published online videos.

### Video assessment

A second-year resident evaluated the videos and their characteristics in the endodontic department. Thirty videos for each search term, which were randomly selected, were reanalyzed by the same observer to evaluate intra-observer agreement at a 2 month interval. The following features of the video were recorded: length of the video, total number of views, likes, dislikes, comments, and days since upload.

The video upload sources were divided into five categories: 1) dentists, 2) specialists, 3) commercials, 4) universities, and 5) others. The videos were divided into two groups depending on the subject: 1) information for dentists and 2) information for laypersons. The video forms were categorized into three groups: 1) real procedure, 2) clinician explanation, and 3) animation. If the video forms overlapped, the main video form was selected.

After categorization of the videos, the viewers’ interests were calculated using the interaction index ([number of likes-number of dislikes] / number of total views × 100%) [5], video power index (VPI) ([view ratio × like ratio / 100]) [10], where view ratio = the number of total views/days since upload and like ratio = [(likes × 100) / (likes + dislikes)].

To assess the content of the videos, the investigator assessed the completeness of each video by numerically scoring (on a scale of 0–2: 0, not mentioned; 1, briefly introduced; 2, introduced in detail) for all six contents of “etiology,” “anatomy,” “symptoms,” “procedure,” “postoperative course,” and “prognosis” with a maximum score of 12 [7].

**Fig. 1:**
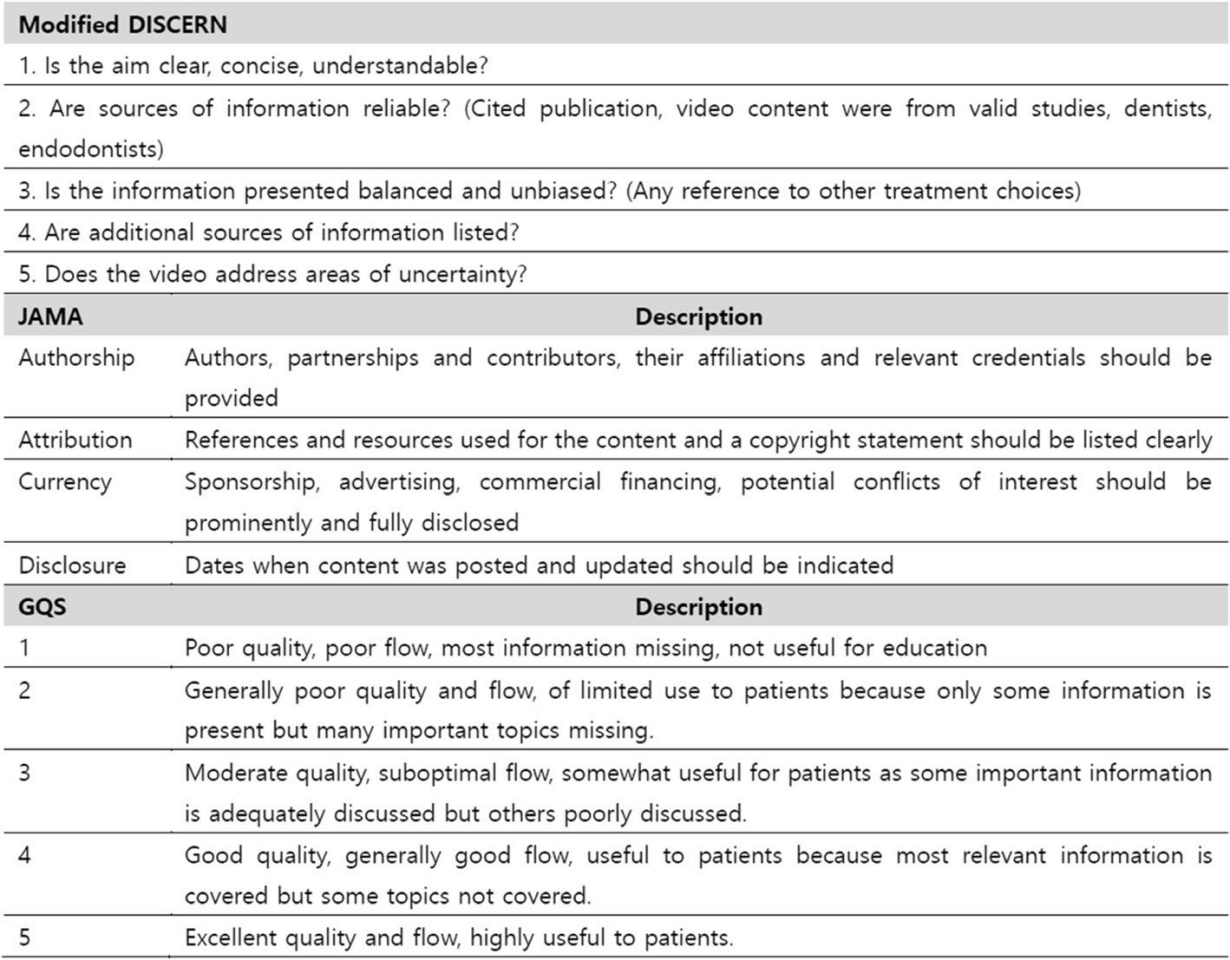
Modified DISCERN, JAMA and GQS. JAMA, *Journal of American Medical Association*; GQS, Global Quality Score

The three evaluation tools used in this study are illustrated in Fig. 1. A modified DISCERN index [11] was selected as a scoring system to evaluate reliability and accuracy using a five-point scale. Each point was allocated for concision, reliability, balance, reference, and uncertainty, with higher scores indicating greater reliability. Similarly, video reliability was evaluated using the JAMA criteria [12]. The JAMA criteria collectively evaluate authorship, attribution, disclosure, and currency. Each criterion was scored (on a scale of 0–1) and the JAVA score was calculated as a total score ranging 0–4, with a maximum score of 4 indicating the highest video reliability.

The quality of educational information was assessed using the GQS [13], with a range of 1–5; where 5 indicates that the quality of information is excellent for the viewer.

### Statistical analysis

SPSS Statistics software (version 16.0; SPSS, Inc., Chicago, IL, USA) was used for statistical analysis. The Shapiro-Wilk and Kolmogorov-Smirnov tests were used to analyze the normality of quantitative data (length of the video, number of total views, likes, dislikes, comments, days since upload, interaction index, VPI, modified DISCERN index, JAMA score, and GQS), and it was shown that the parameters did not represent a normal distribution (*p* < 0.05). The comparison between the two groups for continuous variables with nonnormal distribution was analyzed with the Mann-Whitney test, and the Kruskal-Wallis test was used to compare three or more groups. Pearson’s correlation coefficient was used to examine possible correlations of completeness score, modified DISCERN index, JAMA score, GQS, interaction index, and VPI.

The intraobserver agreement of the rating scores was assessed using intraclass correlation; a value of > 0.8, 0.6–0.8, 0.4–0.6, 0.2–0.4, and < 0.2 represent “excellent” agreement, “very good,” “good,” “questionable,” and “unacceptable,” respectively. Statistical significance was set at *p* < 0.05.

## Results

In this study, a total of 240 videos (120 for each search term) were screened; however, 70 videos of “root canal treatment” and 75 videos of “endodontic treatment” were excluded. The reasons for the video exclusion are listed in Table 1. After screening, 95 videos were analyzed. According to the intraclass correlation, the intraobserver agreement of rating scores between the two evaluation times was 0.856 and 0.810 for “root canal treatment” and “endodontic treatment,” respectively. Descriptive data of the search term, including length of the video, number of total views, likes, dislikes, comments, days since upload, interaction index, and VPI, are shown in Table 2. The average duration of all videos was 320 s. The average number of total views, likes, dislikes, and comments for the entire video was 211,905, 1065, 83, and 160, respectively. The mean number of days since uploading was 895 days. “root canal treatment” videos had a significantly higher number of comments (*p* < 0.05), and “endodontic treatment” videos had a significantly longer duration of videos (*p* < 0.05). However, the number of total views, likes, and dislikes was not significantly different between the search terms (*p* > 0.05).

**Table 1:** Video exclusion reasons for each search term

Reasons	Search term	
	Root canal treatment (***n*** = 70)	Endodontic treatment (***n*** = 75)
No sound or visuals	5	4
Non-English	12	8
Irrelevant	7	4
Longer than 15 minutes	8	24
Duplicated	21	20
Old (uploaded before 2016)	17	15

**Table 2:** Descriptive data by search term

Video Features	Search term		
	Root canal treatment (***n*** = 50)	Endodontic treatment (***n*** = 45)	***p***-value
Duration (second)	273 ± 201	367 ± 229	0.036^*^
Number of total views	269,675 ± 729,554	154,134 ± 353,129	0.170
Number of likes	1195 ± 2717	934 ± 1680	0.911
Number of dislikes	118 ± 383	47 ± 120	0.058
Number of comments	202 ± 449	117 ± 315	0.040*
Days since upload	942 ± 701	848 ± 546	0.994

Values are presented as mean ± standard deviation

Mann-Whitney U test was used

^*^p < 0.05

The characteristics of the video, including the source, subject, and form, are summarized in Table 3. Most of the videos were uploaded by a dentist or specialist (> 70%). The main subject of the video search for “root canal treatment” was information for laypersons (60%), and “endodontic treatment” was for dentists (82%). Finally, the video form of “root canal treatment” is a clinician’s explanation (38%), followed by a real procedure (34%) and animation (28%), and the most video form of “endodontic treatment” is the real procedure (62%).

**Table 3:** Video characteristics

Video characteristics	Search term (%)	
	Root canal treatment	Endodontic treatment
Video source		
Dentist	44	42
Specialist	32	36
Commercial	10	2
University	2	13
Others	12	7
Total	100	100
Video subject		
Information for dentists	40	82
Information for laypersons	60	18
Total	100	100
Video form		
Real procedure	34	62
Clinician explanation	38	7
Animation	28	31
Total	100	100

The viewers’ interaction, completeness, and reliability scores by search terms are presented in Table 4. Evaluations based on video search terms showed that “root canal treatment” had significantly higher etiology and symptom scores (*p* < 0.05), and “endodontic treatment” had a higher interaction index and procedure score (*p* < 0.05). However, there were no significant differences in VPI, completeness score (anatomy, postoperative course, and prognosis), modified DISCERN index, JAMA score, and GQS (*p* > 0.05).

**Table 4:** Interaction index, VPI, completeness score, and reliability scores by search term

Video Features	Search term		
	Root canal treatment (***n*** = 50)	Endodontic treatment (***n*** = 45)	***p***-value
Interaction index	0.75 ± 0.58	1.45 ± 1.52	0.022^*^
VPI	747.2 ± 2727.8	1943.4 ± 9688.5	0.470
Completeness score			
Etiology	0.84 ± 0.84	0.33 ± 0.71	0.001^*^
Anatomy	0.86 ± 0.81	0.84 ± 0.71	1.000
Symptoms	0.58 ± 0.70	0.33 ± 0.64	0.044^*^
Procedure	1.14 ± 0.73	1.53 ± 0.59	0.007^*^
Postoperative course	0.52 ± 0.61	0.40 ± 0.58	0.308
Prognosis	0.38 ± 0.49	0.20 ± 0.41	0.056
Total (max. = 12)	4.32 ± 2.54	3.64 ± 2.07	0.246
Modified DISCERN	1.94 ± 1.24	2.18 ± 1.09	0.333
JAMA	2.54 ± 0.81	2.76 ± 0.74	0.154
GQS	2.88 ± 0.77	2.93 ± 0.69	0.759

Values are presented as mean ± standard deviation

The Mann-Whitney U test was used

*VPI* Video Power Index, *JAMA* Journal of American Medical Association, *GQS* Global Quality Score

^*^p < 0.05

The evaluation of completeness, viewers’ interaction, and reliability scores regarding the video source, video subject, and video form is reported in Tables 5, 6, and 7. According to the video form, the procedure score in the real procedure (*p* < 0.05), etiology, symptoms, and prognosis scores in clinical explanation (*p* < 0.05), and anatomy score in animation (*p* = 0.024) were significantly higher. Videos for dentists had significantly higher procedure scores, while videos for laypersons revealed a higher etiology, anatomy, symptoms, and prognosis score (*p* < 0.05). Furthermore, the total completeness score and interaction index of the videos for laypersons were significantly higher (*p* < 0.05). Uploaded videos by the university had a significantly higher modified DISCERN index (*p* < 0.05), and the JAMA score was significantly higher by commercial (*p* < 0.05). The correlations of all scores that involve total completeness, VPI, interaction index, modified DISCERN index, JAMA score, and GQS are shown in Table 8. The total completeness score showed a high correlation with the GQS (*r* = 0.654) and the modified DISCERN index (*r* = 0.676), and the GQS revealed a high correlation with the modified DISCERN index (*r* = 0.728). The JAMA score showed a low correlation with the modified DISCERN index (*r* = 0.264).

**Table 5:** Completeness score by video source, video subject, and video form

	Etiology	Anatomy	Symptoms	Procedure	Postoperative course	Prognosis
Video source						
Dentist	0.73 ± 0.87	0.85 ± 0.79	0.66 ± 0.73	1.24 ± 0.63	0.51 ± 0.60	0.39 ± 0.49
Specialist	0.50 ± 0.80	0.84 ± 0.72	0.38 ± 0.66	1.50 ± 0.72	0.56 ± 0.62	0.25 ± 0.44
Commercial	0.33 ± 0.52	0.67 ± 0.52	0.00 ± 0.00	1.33 ± 0.52	0.33 ± 0.82	0.00 ± 0.00
University	0.14 ± 0.38	1.00 ± 0.82	0.14 ± 0.38	1.57 ± 0.79	0.00 ± 0.00	0.14 ± 0.37
Others	0.89 ± 0.93	0.89 ± 0.93	0.44 ± 0.73	0.89 ± 0.78	0.33 ± 0.50	0.33 ± 0.50
*p*-value	0.261^a^	0.971^a^	0.052^a^	0.091^a^	0.126^a^	0.249^a^
Video subject						
Information for dentists	0.19 ± 0.48	0.70 ± 0.76	0.21 ± 0.53	1.60 ± 0.56	0.44 ± 0.63	0.14 ± 0.35
Information for laypersons	1.21 ± 0.84	1.08 ± 0.75	0.84 ± 0.72	0.92 ± 0.67	0.50 ± 0.56	0.53 ± 0.51
*p*-value	0.000^b*^	0.017^b*^	0.000^b*^	0.000^b*^	0.437^b^	0.000^b*^
Video form						
Real procedure	0.18 ± 0.49	0.64 ± 0.77	0.20 ± 0.51	1.71 ± 0.46	0.49 ± 0.63	0.11 ± 0.32
Clinician explanation	1.18 ± 0.795	0.95 ± 0.65	1.00 ± 0.69	0.73 ± 0.63	0.41 ± 0.59	0.64 ± 0.49
Animation	0.82 ± 0.095	1.11 ± 0.74	0.46 ± 0.69	1.18 ± 0.67	0.46 ± 0.58	0.32 ± 0.48
*p*-value	0.000^a*^	0.024^a*^	0.000^a*^	0.000^a*^	0.879^a^	0.000^a*^

Values are presented as mean ± standard deviation

^a^Kruskal-Wallis test was used

^b^Mann-Whitney U test was used

^*^p < 0.05

**Table 6:** Total completeness score, interaction index, and VPI by video source, video subject, and video form

	Total completeness score	Interaction index	VPI
Video source			
Dentist	4.39 ± 2.65	1.26 ± 1.62	2695.5 ± 10,419.1
Specialist	4.03 ± 1.99	0.98 ± 0.69	274.9 ± 1208.4
Commercial	2.67 ± 1.37	0.85 ± 0.87	339.2 ± 462.5
University	3.00 ± 0.82	1.35 ± 0.35	70.2 ± 48.1
Others	3.78 ± 2.99	0.59 ± 0.34	330.5 ± 498.7
*p*-value	0.413^a^	0.076^a^	0.186^a^
Video subject			
Information for dentists	3.28 ± 1.73	1.25 ± 1.33	2048.7 ± 8909.9
Information for laypersons	5.08 ± 2.72	0.82 ± 0.85	211.5 ± 406.3
*p*-value	0.002^b*^	0.022^b*^	0.470^b^
Video form			
Real procedure	3.33 ± 1.81	1.32 ± 1.53	2595.9 ± 9980.6
Clinician explanation	4.91 ± 2.41	0.65 ± 0.39	96.4 ± 180.4
Animation	4.36 ± 2.77	1.04 ± 0.78	209.9 ± 399.6
*p*-value	0.046^a*^	0.093^a^	0.219^a^

Values are presented as mean ± standard deviation

*VPI* Video power index

^a^Kruskal–Wallis test was used

^b^Mann-Whitney U test was used

^*^p < 0.05

**Table 7:** Reliability scores by video source, video subject, and video form

	Modified DISCERN	JAMA	GQS
Video source			
Dentist	1.93 ± 1.27	2.49 ± 0.68	2.90 ± 0.74
Specialist	2.41 ± 0.98	2.69 ± 0.64	3.06 ± 0.62
Commercial	1.33 ± 0.82	3.67 ± 0.52	2.50 ± 0.55
University	2.57 ± 1.13	3.29 ± 0.76	3.00 ± 0.58
Others	1.44 ± 1.13	2.00 ± 1.00	2.56 ± 1.13
*p*-value	0.044^a*^	0.001^a*^	0.317^a^
Video subject			
Information for dentists	1.98 ± 0.94	2.67 ± 0.83	2.91 ± 0.58
Information for laypersons	2.16 ± 1.46	2.61 ± 0.72	2.89 ± 0.92
*p*-value	0.544^b^	0.734^b^	0.836^b^
Video form			
Real procedure	1.91 ± 1.00	2.58 ± 0.78	2.89 ± 0.65
Clinician explanation	2.41 ± 1.40	2.55 ± 0.60	3.05 ± 0.65
Animation	2.82 ± 0.91	2.82 ± 0.91	2.82 ± 0.91
*p*-value	0.330^a^	0.242^a^	0.626^a^

Values are presented as mean ± standard deviation

^a^Kruskal-Wallis test was used

^b^Mann-Whitney U test was used

^*^p < 0.05

**Table 8:** Correlation of the scores (r)

	Total completeness score	VPI	Interaction index	Modified DISCERN	JAMA	GQS
Total completeness score	1	− 0.047	− 0.051	0.676^**^	0.064	0.654^**^
VPI	−0.047	1	−0.114	−0.040	0.017	−0.025
Interaction index	−0.051	−0.114	1	−0.088	0.066	−0.043
Modified DISCERN	0.676^**^	−0.040	−0.088	1	0.264^*^	0.728^**^
JAMA	0.064	0.017	0.066	0.264^*^	1	0.163
GQS	0.654^**^	−0.025	−0.043	0.728^**^	0.163	1

Pearson’s correlation coefficient analysis

*VPI* Video Power Index, *JAMA* Journal of American Medical Association, *GQS* Global Quality Score

^**^0.6 ≤ r < 0.8, high correlation

^*^0.2 ≤ r < 0.4, low correlation

## Discussion

Although dentists and laypersons have different purposes for finding information, they are interested in RCTx, commonly performed in dental procedures. Several sources, such as papers, conferences, Internet searches, and social media, provide information on RCTx. YouTube, the second most visited website and can be easily accessed, often provides information to people who want to know about health-related content, and people also upload their opinions. As YouTube content is not evaluated by experts in the relevant field, the reliability and educational quality of health content has become an important issue [14]. The availability and precision of the information on YouTube have been questioned, as the accuracy of the uploaded video cannot be confirmed [15]. Due to its easy accessibility, users can be provided with useful and misleading information. One study reported that 33% of Internet users believed that online medical information is accurate [16]. Therefore, it is necessary to evaluate the videos uploaded to YouTube. As a result, several studies on dentistry, such as bleaching, dental trauma, early childhood caries, clear orthodontic aligners, and cleft lip, have been conducted on YouTube [17–21].

In this study, the search terms were selected from a wide range of terminologies (“root canal treatment” and “endodontic treatment”). This is because if the search term becomes more detailed, the contents related to a specific procedure can be focused.

In the analysis of the descriptive data and characteristics of the videos in this study, the most viewed video was over 5 million, but there were also videos with less than 100 views. Although older videos are generally expected to have higher views, there was no correlation between total views and dates in this study (*r* = v-0.07). The number of likes and dislikes was positively correlated with the total number of views (r > 0.09). There were no differences in the video sources according to the search terms. When YouTube was searched using the term “endodontic treatment,” the main subjects of the videos were dentists (82%), and the majority of the video forms were actual clinical procedures (62%). The average length of the video was also significantly longer than that of videos searched using the term “endodontic treatment.” This means that videos of the search term “endodontic treatment” contain more details on the RCTx procedure than “root canal treatment.”

Several studies have evaluated the popularity of videos using viewing rate, interaction index, and VPI [22, 23]. In this study, the interaction index and VPI were used to assess the popularity of videos. The reason for using the VPI instead of the viewing rate is because it reflects a similar ratio to the viewing rate [24]. The interaction index was significantly higher in the “endodontic treatment” (1.45 ± 1.52) than in the “root canal treatment” (0.75 ± 0.58), which was almost twice as high. This means that viewers are more interested when searching for “endodontic treatment” than “root canal treatment.”

Previous study regarding RCTx content on YouTube [7] reported that the completeness score in the “root canal treatment” group was the highest in the procedure (1.15 ± 0.67), followed by etiology (0.80 ± 0.62), symptoms (0.65 ± 0.81), postoperative course (0.65 ± 0.75), anatomy (0.50 ± 0.69), and prognosis (0.25 ± 0.44). Meanwhile, the score in the “endodontics” group was the highest in the procedure (0.75 ± 0.55), followed by anatomy (0.50 ± 0.61), postoperative course (0.2 ± 0.52), etiology (0.15 ± 0.36), symptoms (0.15 ± 0.49), and prognosis (0.1 ± 0.31). This is consistent with the results of this study, except that the completeness level of the anatomy has been improved. The significant difference in the current study between the two search terms in the completeness of etiology, symptoms, and procedure indicates that “root canal treatment” videos were made mainly for the general public who wondered why they should be treated and what the symptoms were like, and “endodontic treatment” videos were mainly uploaded to dentists who wanted to know the specific treatment procedure.

The average overall completeness score for this study was 4 ± 2.33, showing a low level of approximately 1/3 of the total. This means that the video information related to RCTx is insufficient and may provide YouTube viewers with incorrect information. Furthermore, the completeness scores of the video content by upload source were not significantly different (*p* > 0.05). This was inconsistent with the results of previous studies, in which videos by professionals were more beneficial [25, 26]. Since videos by professionals focus on the procedure itself rather than basic knowledge, it is believed that the completeness of videos by professionals is not greater. Therefore, other evaluation tools are required to analyze professionalism.

Three video evaluation scoring tools were used for objective analysis. The JAMA score focuses on evaluating the reliability of the video, while the modified DISCERN index evaluates both accuracy and reliability. Additionally, the GQS is a gradation of the educational degree of a video. In this study, the mean of the modified DISCERN index by video source was 2.57 ± 1.13, 2.41 ± 0.98, and 1.93 ± 1.27 for universities, specialists, and dentists, respectively, which were significantly higher than those of the non-professional (*p* < 0.05). Videos by professionals were considered to contain clearer references, which had higher accuracy. Furthermore, the JAMA score of the commercial video (3.67 ± 0.52) was the highest, which means that more reliable information was included in the video to enhance the advertisement effect.

In the current study, the correlation between each evaluation tool was analyzed. The total completeness score, modified DISCERN index, and GQS score showed a high correlation (0.6 ≤ r < 0.8). The interaction index and VPI, which indicate the popularity of a video, were not correlated with other video rating tools. This means that even if the video is popular and has many viewers, the quality of the video may not be good (− 0.2 < r < 0.2). However, more studies are needed because few studies have investigated the correlation between video evaluation indices, and the standardized evaluation tool for comprehensive video evaluation remains unclear. A limitation of this study was that only the top 120 videos were analyzed, so not all videos on YouTube regarding root canal treatment could be evaluated. In addition, due to the features of YouTube, many videos are continuously uploaded, and only analysis at a specific point in time is possible. Therefore, an ongoing evaluation of YouTube videos is required. Finally, in addition to YouTube, an additional evaluation of information on social media used by many people, such as Twitter, Facebook, and Instagram, is also necessary, and these previous evaluation tools can be used for analysis.

## Conclusion

Although the accuracy of videos related to RCTx was higher in videos by universities and professionals, the total completeness of YouTube videos was low regardless of the video source. Therefore, it is important for professionals to provide more accurate and reliable videos to reduce the risk of misinformation by viewers. In addition, as there is no standardized index for evaluating YouTube videos, future studies should be directed toward the development of new evaluation tools for more objective and comprehensive video evaluation.

## Data Availability

The datasets used and analyzed during the study are available from the corresponding author upon reasonable request.

## Abbreviations

RCTx: Root canal treatment
JAMA: Journal of American Medical Association
GQS: Global Quality Score
VPI: Video power index
